# Design and Development of a Three-Component Force Sensor for Milling Process Monitoring

**DOI:** 10.3390/s17050949

**Published:** 2017-04-25

**Authors:** Yingxue Li, Yulong Zhao, Jiyou Fei, Yafei Qin, You Zhao, Anjiang Cai, Song Gao

**Affiliations:** 1 The State Key Laboratory for Manufacturing Systems Engineering, Xi’an Jiaotong University, Xi’an 710049, China; yingxueli@stu.xjtu.edu.cn (Y.L.); yafeiqin@stu.xjtu.edu.cn (Y.Q.); zhaoyou628@xjtu.edu.cn (Y.Z.); gaosongg@stu.xjtu.edu.cn (S.G.); 2 School of EMU Application and Maintenance Engineering, Dalian Jiaotong University, Dalian 116028, China; fjy@djtu.edu.cn; 3 Shaanxi Key Laboratory of Nano Materials and Technology, Xi’an University of Architecture and Technology, Xi’an 710055, China; cai_aj@163.com

**Keywords:** table dynamometer, strain type, positional variation, milling force decoupling

## Abstract

A strain-type three-component table dynamometer is presented in this paper, which reduces output errors produced by cutting forces imposed on the different milling positions of a workpiece. A sensor structure with eight parallel elastic beams is proposed, and sensitive regions and Wheastone measuring circuits are also designed in consideration of eliminating the influences of the eccentric forces. To evaluate the sensor decoupling performance, both of the static calibration and dynamic milling test were implemented in different positions of the workpiece. Static experiment results indicate that the maximal deviation between the measured forces and the standard inputs is 4.58%. Milling tests demonstrate that with same machining parameters, the differences of the measured forces between different milling positions derived by the developed sensor are no larger than 6.29%. In addition, the natural frequencies of the dynamometer are kept higher than 2585.5 Hz. All the measuring results show that as a strain-type dynamometer, the developed force sensor has an improved eccentric decoupling accuracy with natural frequencies not much decreased, which owns application potential in milling process monitoring.

## 1. Introduction

Many technologies have been applied to monitor machining processes [1]. Cutting force is an important indicator to evaluate inconspicuous tool wear, cutting vibrations, chip formation and machining errors during machining processes [2,3,4,5]. As one category of cutting forces, milling force is one of the most widely studied features of any milling procedure. Various measuring methods have been tried to obtain its real-time value [6]. With their excellent performance advantages, piezoelectric dynamometers were the most extensively employed instruments [7,8]. However, electrical discharge is necessary for the matching charge amplifier, and the discontinuous working time makes measuring over a relatively longer machining duration inconvenient; in addition, the high price has restricted their industrial applications. Strain-type dynamometers with low manufacturing cost are another feasible milling force measurement method. Based on the strain effect, rotating dynamometers mounted on main spindle and table force sensors placed between the workbench and workpiece were both developed. Because of their few mounting constraint requirements, various types of rotating dynamometers have been proposed too [9,10,11]. However, considering the difficulties in prolonging the power supply time of their wireless network modules and as well as enhancing the sensor bandwidth limited by the stiffness of the milling spindle [12,13], table force sensors are mainly studied at this time.

During the machining process, when a workpiece is fastened on a table dynamometer, a milling tool continually cuts through it, which causes the force bearing point on the table dynamometer to change. Since in a sensor calibration process, a decoupling matrix is obtained from one fixed force bearing point, the matrices gauged on the different force bearing points for a table force sensor may not be unified without some milling position compensation. Moreover, the contradiction of sensitivity and stiffness of the dynamometer increases the complexity of the structural design. In the case the differences of cross couplings were ignored, a dynamic force sensor with octagonal rings was proposed by Shaw [14], and its principal components in each measuring circuit showed little variation as the force bearing point moved (≤1.3%) [15]; when cross couplings were taken into account, the author put forward a table dynamometer with a cross beam structure, which reduced the static decoupling errors between measured forces and standard inputs to be not more than 4.87% [16], but its lowest natural frequency only reached 920 Hz; Zhao and his team have developed a three-component milling force measuring platform, whose minimum resonance frequency exceeded 9 KHz [17,18], whereas, without the torque compensation matrix associated with the milling position, its maximum decoupling error in machining processes was no less than 8% [19]. Although there are not many related studies, when both principal and cross coupling eccentric force components are taken into account, a strain type force sensor capable of reducing their output errors is needed. Besides, the natural frequency of the sensor also must be considered.

A three components table milling force sensor with strain gauges is proposed in this paper. Eight parallel sensitive beams were applied to promote its natural frequencies, and then the vertical beam structure was transformed to reduce the influence of eccentric force on the measuring circuit output. A stress concentration method of section change was finally applied in its fabrication. Lastly, the sensor decoupling performance was confirmed via static calibration tests and dynamic milling experiments.

## 2. Sensor Design and Fabrication

### 2.1. Sensor Structure Design

#### 2.1.1. The Initial Model for Sensor Structural Design

**Figure 1:**
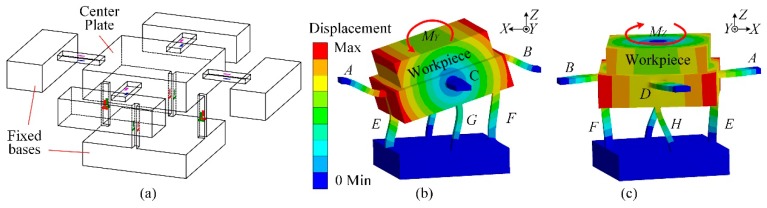
(**a**) Original design of the three components force sensor; (**b**) Sensor deformation under *M_Y_*; (**c**) Sensor deformation under *M_Z_*.

Because a beam with tension and compression deformation has high stiffness along its axial direction, a three component force sensor with parallel beam structure is tentatively put forward as shown in Figure 1a. The strain gauges with resistance value *R* are all bonded at the midpoint of the beam surfaces, where they mainly measure the force components having the same orientation as the beam axial directions where they are pasted.

In model abstraction, the sensor center plate and the workpiece mounted on it are regarded as a rigid body. If an external moment in *Y* direction (*M_Y_*) is applied on the rigid body, its rotating shaft is basically coincident with the axes of beam *C* and *D*. Owing to the symmetry of the sensor structure, the axes of beam *A* and *B* are coaxial with rotating shaft under *M_X_*. Besides, as shown in Figure 1c, the sensor rotates around sensor central axis in the *Z* direction under *M_Z_*. The three rotation axles intersect at one point (*O*). Based on the theorem of translation of a force, an eccentric force imposing on the rigid body is equivalent to a force with the same magnitude and direction acting on point *O*, and superposing an additional moment *M* relative to this point. In order to obtain the same sensor output values without caring whether or not it is an eccentric load, the outputs derived from the input of the additional moment *M* had better be zero. Therefore, all the following discussions focus on the analysis of the output signals under *M*. As *M* is further resolved into *M_X_*, *M_Y_* and *M_Z_* as listed in Table 1, each of them will be separately investigated in the following sections. The less output values are measured under these additional moments, the more eccentric influences are reduced by the dynamometer.

#### 2.1.2. Sensor Output under Additional Moment

**Figure 2:**
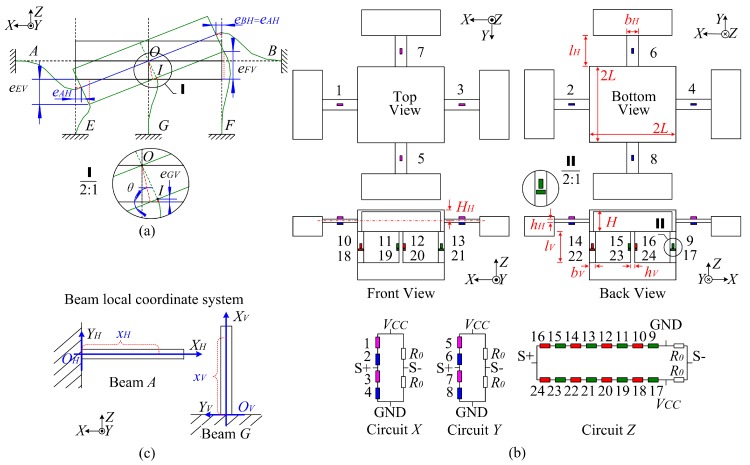
(**a**) Sensor deformation under *M_Y_*; (**b**) Sensor measuring circuits; (**c**) Beam local coordinate systems.

Owing to the symmetry of the sensor structure, the output signals under *M_X_* are mostly ignored in the following discussions. When *M_Y_* is applied, as the rotation angle (*θ*) is quite small, according to Figure 2a and Equation (1), the length variation in beam *G* (*e_GV_*) is ignored, and the amounts of elongation in beam *E* (*e_EV_*) and shortening in beam *F* (*e_FV_*) are approximately equal. There is almost no *Z* directional resultant force applied on the sensor center plate by vertical beams. Thus, under *M_Y_*, the stresses along the axes of the vertical beams are given in Equation (2). According to the linear relation between the resistance variation of a strain gauge (Δ*R*) and the stresses (*σ*) in its corresponding sensitive regions (Δ*R* = *KσR*/*E*, where *K* and *R* represent the sensitivity coefficient and resistance value of a strain gauge, and *E* is the Young modulus of the beam material), the strain gauges 9–16 on beam *E* and *F* are connected in series as one arm of a Wheastone bridge circuit to eliminate *M_Y_* impact. The strain gauges 9–16 and 17–24 are serially connected in the adjacent bridge arms to cancel a part of the temperature effects. The measuring circuit *Z* is shown in Figure 2b, and *R*_0_ is a fixed resistance.

(1) $$\begin{array}{ccc} e_{GV}=OI\times \tan\frac{\theta }{2}\times \sin\theta , & e_{EV}=\left(L-OI\times \tan\frac{\theta }{2}\right)\times \tan\theta & e_{FV}=\left(L+OI\times \tan\frac{\theta }{2}\right)\times \sin\theta ; \end{array}$$

(2) $$\begin{array}{cc} \sigma \left(E,M_{YV}\right)=-\sigma \left(F,M_{YV}\right)=\frac{Ee_{EV}}{l_{V}}, & \sigma \left(G,M_{YV}\right)=0; \end{array}$$

where *OI* is the length of the line segment between the rotation center *O* and its *Z* directional projection (point *I*) on the bottom of the central platform; *L* is the half length of the sensor center plate; *σ*(*i*, *M_YV_*) is the bending surface axial stress on beam *i* under *M_YV_*; *M_YV_* is the part of *M_Y_* distributing on vertical beams; *l_V_* is the length of the vertical beams.

As depicted in Figure 2a, the elongations of beam *A* and *B* in *X* direction (*e_AH_* and *e_BH_*) under *M_Y_* are equal, which means that their corresponding axial forces do not influence the sensor center plate. Thus, the top and bottom surface stresses of the horizontal beams under *M_Y_* are similar to the model abstracted from a cross beam sensor except applied with a different value of the external moment, as expressed in Equation (3) [20].

(3) $$\sigma \left(A,M_{YH}\right)=-\sigma \left(B,M_{YH}\right)=\mp \frac{3}{4b_{H}h_{H}{}^{2}}\frac{l_{H}{}^{2}+3Ll_{H}-3x_{H}\left(L+l_{H}\right)}{\left(l_{H}{}^{2}+3Ll_{H}+3L^{2}\right)}M_{YH}$$

where *σ*(*, *M_YH_*) is the bending surface stresses of beam *A* and *B* under *M_YH_*; *M_YH_* is the part of *M_Y_* distributing on beam *A* and *B*; *x_H_* is the variable in local coordinate system of the beam, as shown in Figure 2c; *b_H_*, *h_H_* and *l_H_* are the width, thickness and length of the horizontal beams.

Referring to the measurement circuits in the cross beam sensor, the strain gauges on the same horizontal beams are connected in series as one bridge arm to offset the bending outputs under *M_Y_*. By connecting strain gauges of different beams on the adjacent arms of a Wheastone bridge circuit, the resistance variations generated by *e_AH_* and *e_BH_* are eliminated. The measuring circuit *X* is drawn in Figure 2b. When *M_X_* is applied on the sensor, beam A and B both twist. Because little influences on the sensitive areas are produced by torsion and warping stresses, the output of circuit *X* under *M_X_* can also be neglected.

When *M_Z_* is applied on the workpiece, with strain gauges bonded on the bending neutral planes of the horizontal beams, the output of measuring circuit *X* is almost not affected. Based on Equation (4), with the locations of *x_V_* ≈ *l_V_*/2 in beam local coordinate systems, the stresses of strain gauges 9–24 nearly do not change, and the output of circuit *Z* is basically not affected by *M_Z_* either:

(4) $$\begin{array}{cc} \sigma \left(i,\;M_{ZV}\right)=\pm \frac{3\left(l_{V}-2x_{V}\right)}{4b_{V}h_{V}{}^{2}l_{V}}M_{ZV} & i=beam\;E,F,G,H; \end{array}$$

where *σ*(*i*, *M_ZV_*) is the bending surface stress on beam *i* under *M_ZV_*; *M_ZV_* is the part of *M_Z_* distributing on the vertical beams; *x_V_* is the variable in local coordinate system of the beam, as shown in Figure 2c; *b_V_* and *h_V_* are the width and thickness of the vertical beams.

#### 2.1.3. Beam Structural Adjustment Based on *M_Y_*

All the analyses in the last section is based on the Euler–Bernoulli beam theory. In a practical design, to avoid the weakest torsional stiffness in *Z* direction pulling down the resonant frequency of the sensor, the width of the horizontal beam (*b_H_*) is enlarged, which exceeds the slender beam assumption. Therefore, its bending stresses are influenced by the cross section angle of the beam end. Because of the symmetry of the sensor structure, and as well as the small width of the strain gauge, the outputs of measuring circuit *X* and *Y* are not much affected by *M_Z_*. However, when *M_Y_* is applied, there is a difference (Δ*σ*(*θ*)) of the stress magnitude between the top and bottom surfaces of beam *A* and *B* compared with Equation (3). The objective function *σ_r_* = ( *σ*_1_ + *σ*_2_ ) − ( *σ*_3_ + *σ*_4_ ) = 2Δ*σ*(*θ*) proportional to the output of circuit *X* cannot be completely compensated, where *σ_i_* represents the stress on the sensitive area of strain gauge *i*. As the distance between the axis of the horizontal beam and the top surface of the workpiece (*H_H_*) impacts the rotating stiffness of the sensor, to find out the proper angle *θ* having no effect on the horizontal beam stresses, the values of *θ* and *σ_r_* are solved by taken *H_H_* as the variable in ANSYS simulation. The sensor conditions for simulation are listed in Table 2. The results are tabulated in Table 3.

**Figure 3:**
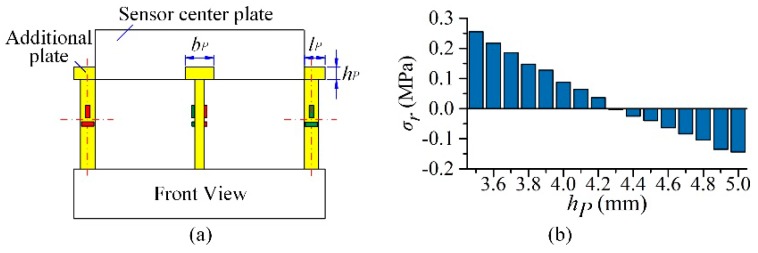
(**a**) New structure of vertical beams; (**b**) *σ_r_* variations with *h_P_* changing.

It is observed that though the absolute value of *σ_r_* decreases as *θ* rises, *σ_r_* cannot be fully eliminated, which means that the *Y* axial rotating stiffness still limits the section deformation of the horizontal beams. If *F_X_* has the same magnitude with the force component generating *M_Y_*, *M_Y_* will bring about 2.14–2.64% interferences to the measuring circuit *X* in this sensor structure. In order to reduce the sensor rotation stiffness in *Y* direction, a vertical beam with lower axial stiffness is demanded. Thus, a new structure of the vertical beam is proposed as shown in Figure 3a, and the additional serially connected plate decreases its axial stiffness without changing both of the position and variation rules of the strain sensitive areas. By adjusting the thickness (*h_P_*) of the additional plate, the stiffness of the beam is varied. With 8 mm of *H_H_*, the base area of 9 mm × 12 mm (*l_P_* × *b_P_*) of the additional plate, and other dimensions unchanged, the value of *σ_r_* declines with *h_P_* reduction, as plotted in Figure 3b. When *h_P_* is 4.3 mm, the minimum absolute value of *σ_r_* reaches −2.31 × 10^−3^ MPa, which is the desired structure for the sensor. Therefore, with the structural adjustment of sensor vertical beams, there is almost no interference brought by *M_Y_* in circuit *X*.

### 2.2. Sensor Fabrication

**Figure 4:**
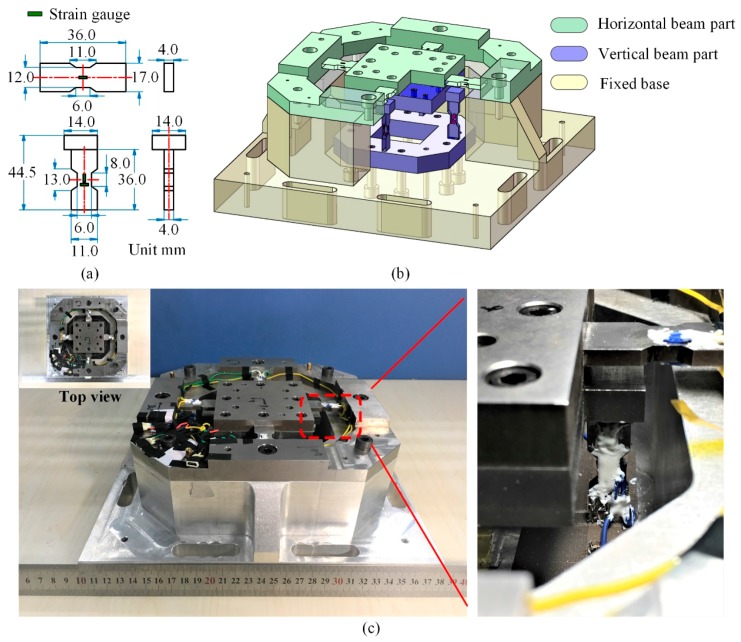
(**a**) Beam dimensions for the fabricated sensor; (**b**) Sensor assembly diagram; (**c**) The photos of the fabricated sensor.

Since the sensor stiffness is decreased by the beam structural adjustment, by using variable cross sections on the beams, the width of the beam is increased to enhance the sensor resonant frequencies. The fabricated dimensions of the sensor beams are shown in Figure 4a, and the size of sensor center plate and the value of *H_H_* are unchanged. With damping ratio 0.01, by using ANSYS simulation, the measuring range of the sensor is approximately ±800 N for each component. Within the measuring range, the maximum displacement of the sensor center plate in the steady state response simulation is 9.59 μm, when the force is applied on the corner of the center plate. In order to reduce the sensor weight and bring convenience to strain gauge bonding, the main body of the sensor is composed of two parts, one containing horizontal beams and the other including vertical beams, which are both made of AISI630 (17-4 PH) stainless steel, and the fixed base of the sensor is made of 7075-T6 aluminum alloy. Its assembly diagram is shown in Figure 4b. The measuring circuits depicted in Figure 2b are used. TP-3.8-1000 (1 KΩ) semiconductor strain gauges (produced by Tianguang Sensor, Bengbu, China) are selected, and resistances *R_0_* in circuit *X*, *Y* and *Z* are 5.1 KΩ, 5.1 KΩ and 20 KΩ, respectively. Silicon rubber is painted on to protect the strain gauges. The photos of the fabricated sensor are shown in Figure 4c.

## 3. Experimental Results and Discussion

### 3.1. Static Calibration Test

**Figure 5:**
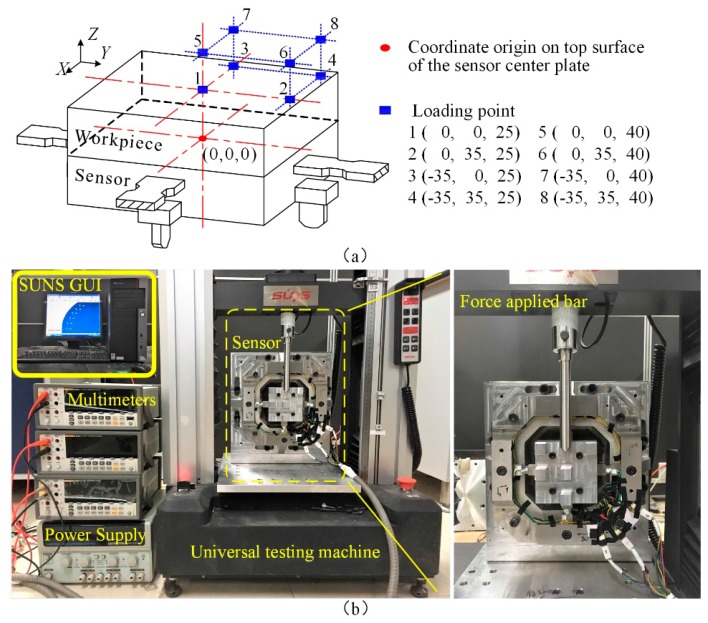
(**a**) Eight force bearing points 1–8 for static calibration test; (**b**) Setups for static calibration test.

Static calibration is performed to determine the static relationship between the tri-axial force and the sensor output. To evaluate the eccentric forces influencing on the sensor outputs, eight force bearing points with different positions on the workpiece were selected in the static calibration test. The schematic diagram of the eight points 1–8 is shown in Figure 5a, the coordinate origin is the midpoint on the top surface of the center platform. As shown in Figure 5b, the uniderectional force was applied by an electro-mechanical universal testing machine (type UTM6104, SUNS Technology, Shenzhen, China), and a DC regulated power supply (type GPS-3303C, GWINSTEK, New Taipei City, Taiwan) and three high accuracy digital multimeters (type 8846A, FLUKE, Everett, WA, USA) were utilized to record the data of sensor output. To clearly distinguish the differences of the sensor output signals, three amplifiers (type PGA308, TI, Dallas, TX, USA) were connected to the sensor output. The amplification coefficients are separately 156.40, 157.90 and 188.50 for measuring circuit *X*, *Y* and *Z*. The supply voltage of each Wheatstone bridge circuit in the sensor is 4.096 V. In the calibration process, the standard forces are linearly varied from 0 to 800 N with a step length of 100 N. They increase first step by step in loading processes, and then decrease with the same step length in unloading procedures. At each test point, every force component is measured 3 times with loading and unloading process, and their average values are used in the following comparisons.

#### 3.1.1. Output Errors of the Principal Components in Each Circuit

**Figure 6:**
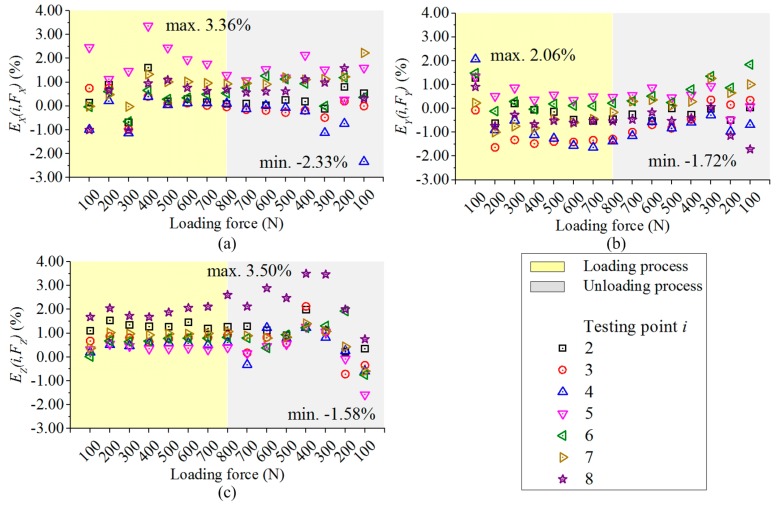
(**a**) Output errors of circuit *X* under *F_X_*; (**b**) Output errors of circuit *Y* under *F_Y_*; (**c**) Output errors of circuit *Z* under *F_Z_*.

The output signals of point 1 are taken as the reference values. The differences of the principal components in the outputs of the measuring circuits between point 1 and the other positions under the same force input are compared based on Equation (5), and their values are plotted in Figure 6.

(5) $$\begin{array}{ccc} E_{j}(i,F_{j})=\frac{U_{j}(i,F_{j})-U_{j}(1,F_{j})}{U_{j}(1,F_{j})} & i=2,3,...,8; & j=X,Y,Z; \end{array}$$

where *E_j_*(*i*, *F_j_*) is the relative error derived on point *i* in circuit *j* under force component *F_j_*; *U_j_*(*) denotes the voltage value of measuring circuit *j*.

As illustrated in Figure 6, during both loading and unloading procedures, the differences of the mainly measured force components are not more than 3.36%, 2.06% and 3.50% in circuit *X*, *Y* and *Z*, respectively. This demonstrates that though the force bearing point changes, the output errors of the principal components in each measuring circuit are reduced by the dynamometer.

#### 3.1.2. Output Errors of the Cross Couplings in Each Circuit

**Figure 7:**
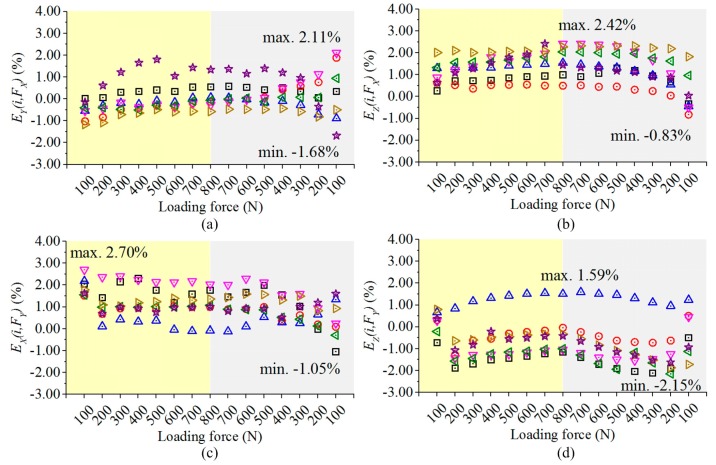

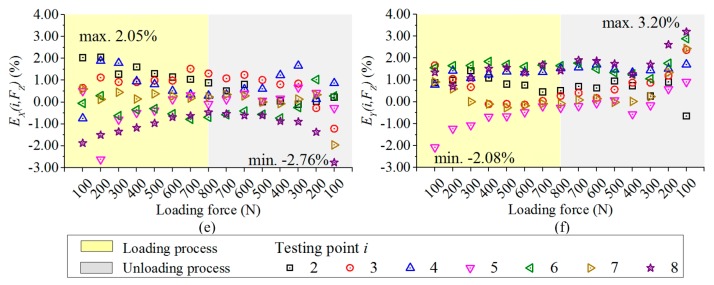
(**a**) Output errors of circuit *Y* under *F_X_*; (**b**) Output errors of circuit *Z* under *F_X_*; (**c**) Outputs error of circuit *X* under *F_Y_*; (**d**) Outputs error of circuit *Z* under *F_Y_*; (**e**) Output errors of circuit *X* under *F_Z_*; (**f**) Output errors of circuit *Y* under *F_Z_*.

The cross coupling is assessed by the ratio of a signal produced by the interferential force component to the primary output of the same measuring circuit. To quantify the differences of the interference signals, the principal components in each measuring circuit on testing point 1 are taken as the references to calculate all of the cross couplings, and their variations are figured out based on Equation (6). By using the same legend as plotted in Figure 6, and the results are shown in Figure 7

(6) $$\begin{array}{l} \begin{array}{cc} E_{j}(i,F_{k})=\frac{U_{j}(i,F_{k})-U_{j}(1,F_{k})}{U_{j}(1,F_{j})} & i=2,3,...,8; \end{array}\\ \begin{array}{ccc} \;j=X,Y,Z; & k=X,Y,Z; & j\neq k, \end{array} \end{array}$$

where *E_j_*(*i*, *F_k_*) stands for the error of cross coupling obtained in measuring circuit *j*, when *F_k_* is applied on testing point *i*. As plotted in Figure 7, during unloading and loading processes, the variations of the cross couplings among different loading points do not exceed 2.76%, 3.20% and 2.42% in *X*, *Y* and *Z* circuits, respectively. The results indicate that the variations of the cross-coupling components in measuring circuits caused by eccentric forces are also decreased by the developed force sensor.

#### 3.1.3. Static Force Decoupling

Since the measuring ranges of the three components in the sensor are alike, resultant forces composed of three equal components are used to verify the decoupling performance of the dynamometer. The recorded signals from each measuring circuit gauged at the same testing point and under the identical force are superposed together, which constitute the output voltages (*U_X_*, *U_Y_* and *U_Z_*) in Equation (7):

(7) $$\begin{array}{l} U_{OUT}=DF+U_{0}\\ \left[\begin{array}{c} U_{X}\\ U_{Y}\\ U_{Z} \end{array}\right]=\left[\begin{array}{ccc} 1.72\times 10^{-3} & 2.14\times 10^{-5} & 2.63\times 10^{-5}\\ 4.70\times 10^{-5} & 1.73\times 10^{-3} & 1.18\times 10^{-5}\\ 3.57\times 10^{-5} & 5.57\times 10^{-5} & 1.67\times 10^{-3} \end{array}\right]\left[\begin{array}{c} F_{X}\\ F_{Y}\\ F_{Z} \end{array}\right]+\left[\begin{array}{c} 3.50\times 10^{-3}\\ -1.28\times 10^{-3}\\ -9.47\times 10^{-3} \end{array}\right] \end{array}$$

where *U_OUT_* is the matrix of output voltages involving three-circuit outputs (*U_X_*, *U_Y_* and *U_Z_*), and the unit is V; *U_0_* is the zero offset, and its unit is also V. The decoupling matrix (D) is formed with the slope coefficients derived by the least squares line fitting of the measured data.

**Figure 8:**
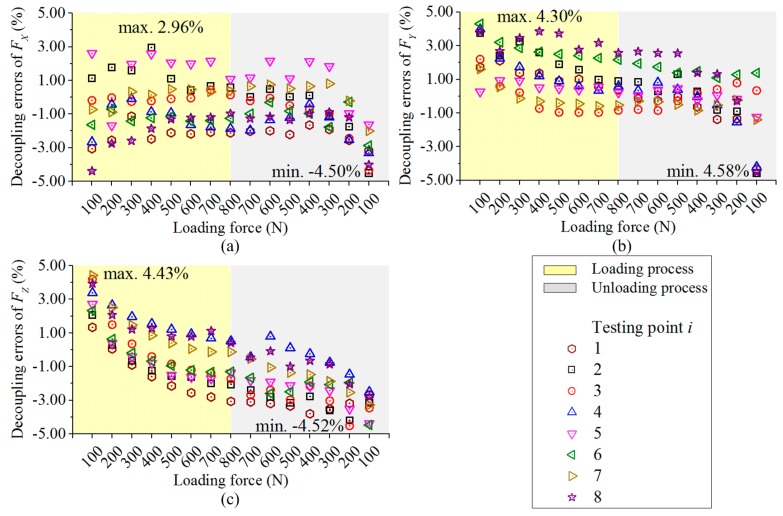
(**a**) Decoupling errors of *F_X_*; (**b**) Decoupling errors of *F_Y_*; (**c**) Decoupling errors of *F_Z_*.

Based on Equation (7), the three force components measured by the dynamometer are figured out, and their decoupling errors compared with the standard forces (100 N–800 N) of the universal testing machine are depicted in Figure 8. As it is presented in Figure 8, when both loading and unloading data are taken into account, the deviations between the measured force components and the standard input are not larger than 4.50%, 4.58% and 4.52% for *F_X_*, *F_Y_* and *F_Z_*, respectively. The maximal difference of their resultant forces is −4.07%, which is obtained under input force components of 100 N at testing point 2 in unloading process. The static deviations are acceptable when the force bearing point moves within the calibration range.

The linearity error, hysteresis error and repeatability error derived on each loading point are listed in Table 4 [21]. The fitting curves to calculate linear errors are based on Equation (7). The maximal absolute values of linearity errors, hysteresis errors and repeatability errors separately are 1.55%, 0.58% and 0.82%, which also shows a good static performance for three-component force measurement.

### 3.2. Milling Experiment

Milling experiment of the sensor was carried out to verify its dynamic performance in the actual machining operation.

#### 3.2.1. Resonant Frequency Identification

**Figure 9:**
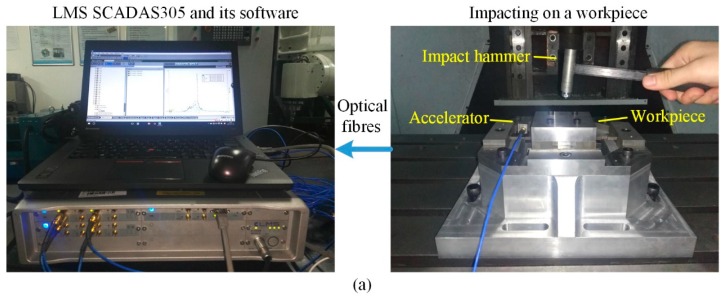

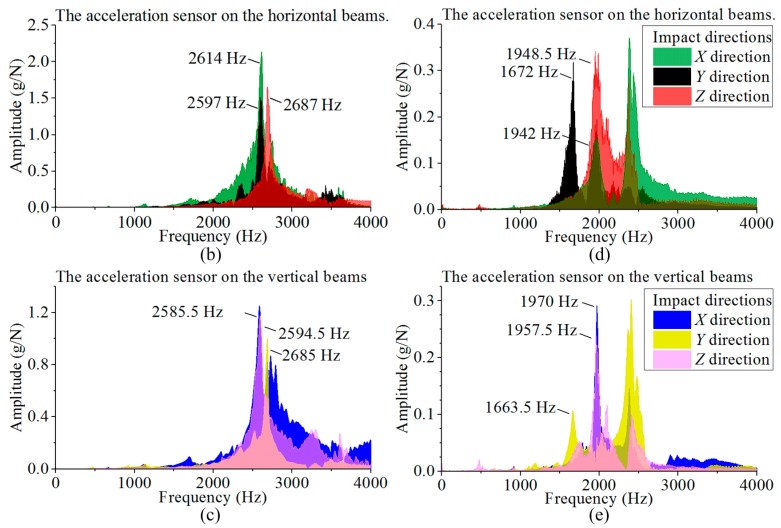
(**a**) Impact testing for the developed sensor; (**b**) Impact response with accelerator on the horizontal beam; (**c**) Impact response with accelerator on the vertical beam; (**d**) Workpiece impacted with accelerator on the sensor horizontal beam; (**e**) Workpiece impacted with accelerator on the sensor vertical beam.

To implement the resonant frequency measurement, as shown in Figure 9a, a tri-axial piezoelectric accelerator (type 356A66, PCB, Depew, NY, USA) is stuck on the sensitive areas of the beams, and impact hammer (type 086E80, PCB) is used to tap on the sensor center plate or workpiece. These signals are collected in a mobile LMS SCADAS305 data acquisition cabinet (SIEMENS, Leuven, Belgium) and processed by software of LMS Test Lab.

As plotted in Figure 9b,c, the lowest sensor natural frequency is 2585.5 Hz. With an 88 × 88 × 20 mm^3^ AISI1045 steel workpiece, the minimal resonant frequency is 1663.5 Hz, as shown in Figure 9d,e.

#### 3.2.2. Milling Test and Results

**Figure 10:**
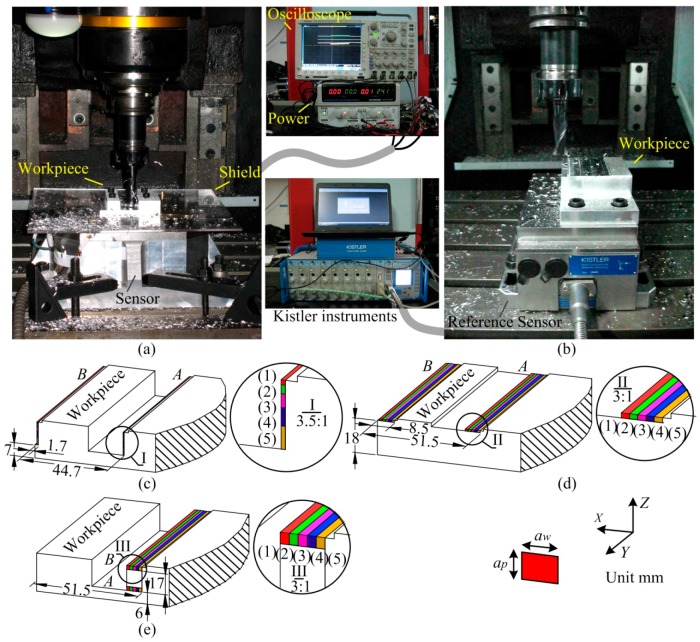
(**a**) The developed sensor in milling experiment; (**b**) The referring dynamometer in milling test; (**c**) Milling positions of test group 1; (**d**) Milling positions of test group 2; (**e**) Milling positions of test group 3.

**Figure 11:**
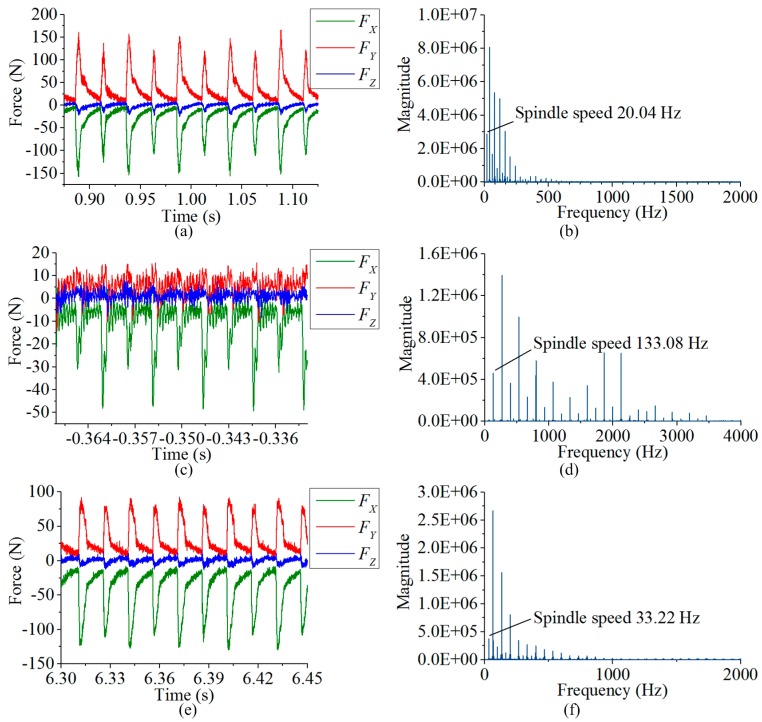
(**a**) Decoupling forces of test 1 (5) *A* from developed sensor; (**b**) FFT of *F_X_* in test 1 (5) *A* from developed sensor; (**c**) Decoupling forces of test 2 (5) *A* from developed sensor; (**d**) FFT of *F_X_* in test 2 (5) *A* from developed sensor; (**e**) Decoupling forces of test 3 (5) *A* from developed sensor; (**f**) FFT of *F_X_* in test 3 (5) *A* from developed sensor.

The dynamic milling experiment was implemented in a three-axis CNC vertical milling machine (type BCH850), and two edges milling tool with the diameter of 16 mm (type GM-2E-D16.0, ZCCCT, Zhuzhou, China) was used. The output signals are saved by an oscilloscope (type MSO 4104, Tektronix, Beaverton, OR, USA) configured with sampling rate of 25 KHz. As shown in Figure 10a,b, the milling operation was first implemented on the developed sensor, and then with the same machining parameters, the measured data on a commercial milling force sensor (type 9265B, Kistler) was also recorded as a reference. All the milling operations were carried out along positive *Y* axial direction in down milling mode. Three groups of experiments were performed on the developed sensor. In each test group, a pair of milling subtests (marked with *A* and *B*) were implemented with the identical process parameters but different machining positions, as Figure 10c–e show, and their operation parameters are listed in Table 5, where *a_p_*, *a_w_*, *v_f_* and *n* separately stand for the axial cutting depth, radial cutting width, feeding rate and spindle rotation speed. Based on Equation (7), the waveforms recorded from oscilloscope are processed in Matlab. Some examples of decoupling forces (test 1 (5) *A*, test 2 (5) *A* and test 3 (5) *A*) are plotted in Figure 11, and the Fourier transformations of their *F_X_* values are also drawn on the left side of the graph. The chatter signals in Figure 10c are generated by the resonance of the milling machine table, which are around 2000 Hz as shown in Figure 10d.

**Figure 12:**
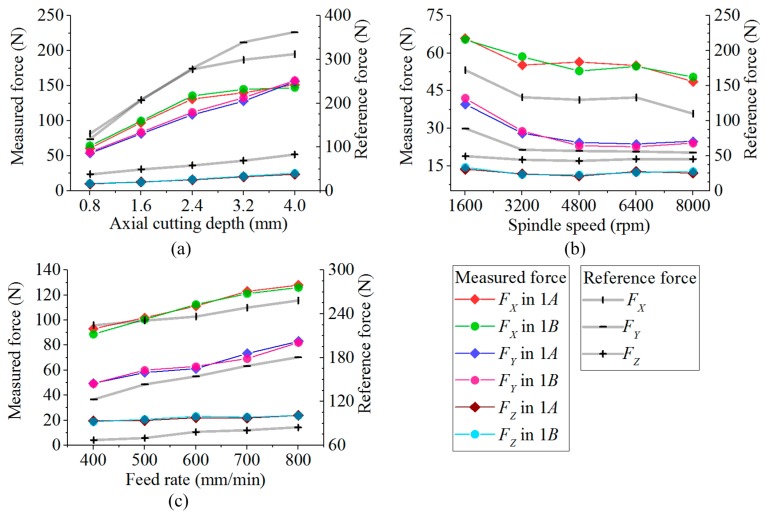
(**a**) Measured forces and reference results of test group 1; (**b**) Measured forces and reference results of test group 2; (**c**) Measured forces and reference results of test group 3.

In every group, the force data in stable milling state are picked out. Based on these data, the peak to peak amplitudes of force components derived from each spindle cycle are averagedas expressed in Equation (8). The average values of measured forces and reference results gauged by Kistler device are all depicted in Figure 12. Though neither of the bearing plates in these two dynamometers have enough areas to put one sensor on the other, to validate the usability of the data obtained from the developed dynamometer, these data still had to be roughly compared with the reference values. Their cross correlation coefficients (*R*) are listed in Table 6.

(8) $$\begin{array}{ccc} F_{j}{\left(i\right)}_{P-P}=\frac{1}{N}\sum_{c=1}^{N}F_{j}{\left(i,c\right)}_{P-P} & i=1\left(1\right)A,1\left(2\right)B,...,5\left(5\right)B; & j=X,Y,Z, \end{array}$$

where *N* represents the number of the spindle cycles involved in the force stable period; *F_j_*(*i*, *c*)*_P_*_-*P*_ denotes the peak to peak amplitude derived in the *c*th spindle cycle from milling position *i*; *F_j_*(*i*)*_P_*_-*P*_ is the average value of *F_j_*(*i*, *c*)*_P_*_-*P*_.

From Table 6, it illustrates that the measured force components keep good linear correlations with the reference arrays. Thus, the measured data are able to be used in the following comparisons. Taking the data in subtest *A* as a new reference, the differences of the measured forces between subtests *A* and *B* are tabulated in Table 7.

According to Table 6, the maximal deviations between subtests *A* and *B* are separately −6.29%, 6.03% and 6.21% for *F_X_*, *F_Y_* and *F_Z_*. The eccentric errors of measured forces are reduced as expected by the developed dynamometer.

### 3.3. Discussion

The performances of the previously developed strain-based dynamometers are summarized in Table 8 and Table 9.

**Figure 13:**
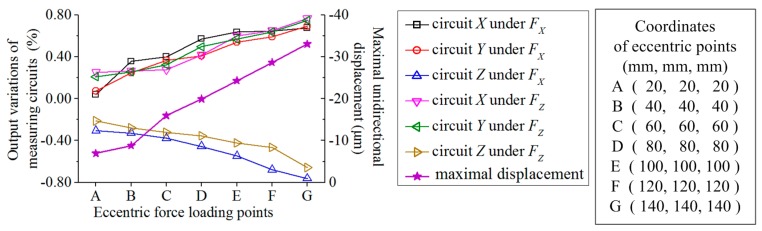
Simulation results based on different eccentric force loading points.

Though the influences of eccentric forces on the principal components in measuring circuits are reduced significantly by the dynamometer with octagonal rings, the decoupling components grow fast with the eccentric offset of the force bearing point; the resonance frequency is largely promoted by the force sensor with paralleled vertical beams, but a decoupling matrix of eccentric moment is necessary, or else its decoupling errors are not satisfied for milling force measurement. Considering decoupling accuracy and resonance frequency together, it shows that the developed force sensor has a more balanced performance than the previous works. In the entire experimental procedure, the variation range of the loading position does not exceed (44, 44, 20), which corresponds to a workpiece with dimensions of 88 × 88 × 20 mm^3^. According to the results simulated by ANSYS plotted in Figure 13, when the eccentric offset is beyond the experimental range, the eccentric influence does not increase quickly with the eccentric range growth. To draw Figure 13, the stresses on the midpoints of each sensitive region are used to replace the signals of measuring circuits, and the midpoint on the top surface of the sensor center platform is taken as the coordinate origin. When a unidirectional force of 800 N is applied on the point with coordinate of (140, 140, 140), the variations of the circuit outputs are all within 0.77%. However, with the eccentric offset of the loading point increasing, the total displacement of the sensor center plate raises as well. When a force with three components of 800 N is applied, the maximal unidirectional displacement is plotted based on the left vertical axis of Figure 13. When the eccentric range reaches (80, 80, 80), the static displacement in *X* direction has achieved 19.93 μm, which may bring effect to the accuracy of cutting operation. Moreover, as the natural frequency is inversely proportional to the square root of mass, when the workpiece rises, the resonant frequency of the measuring system will be reduced. Therefore, it is a constant goal to enhance sensor natural frequencies, increase its sensitivities and improve decoupling accuracies. One of the advantages of the developed sensor is that all the above discussed eccentric offsets of the loading points are not limited by the positions of the elastic beams, which means that some of the force loading points have already exceeded the positions of the elastic beams. If the structure of the developed sensor could be miniaturized, it might be used as a three-component force measuring cell to constitute a dynamometer with a larger plate, but this assumption is also based on the improvement of sensor natural frequencies and output sensitivities.

## 4. Conclusions

A three-component milling dynamometer with eight parallel beams was proposed to reduce the output errors caused by different loading positions in dynamic milling processes. With sensor structure design, sensitive area selection and measuring circuit connection, the influences on sensor outputs caused by eccentric milling force are decreased.

Comparisons of test results with the same load input when operated on different positions of the workpiece were made. In a static calibration test, the differences of the principal output components in each measuring circuit are not more than 3.50%, and those of the cross-coupling components do not exceed 3.2%. The maximum decoupling deviation between the measured forces and the standard inputs is 4.58%. In dynamic milling processes, with identical machining parameters, the differences of measured force components in different cutting positions are within 6.29%. Moreover, owing to the application of parallel beams, with a center plate of 88 × 88 × 25 mm^3^ and a workpiece of 88 × 88 × 20 mm^3^, the lowest resonant frequency of the sensor still reaches 1633.5 Hz. Our experimental results demonstrate that the developed dynamometer is appropriate for dynamic force measurement when milling tool cuts in different places of the workpiece. The sensor has potential application value for milling force monitoring.

Future work will still focus on research of enhancing the sensor stiffness and decoupling accuracy, such as with a new beam structure design, or a method of milling position compensation, etc. which are all good approaches to obtain a better performance for the milling force sensor in practical applications. Besides, measurement methods of dynamic performance also need to be studied. As the bandwidth and volume of the dynamometer increase, it is necessary to develop an appropriate dynamic force calibration device.

## Figures and Tables

**Table 1:** Decomposition of an additional moment.

	Additional Moment *M*					
Moment decomposition	*M_X_*		*M_Y_*		*M_Z_*	
Applied force component	*F_Y_*	*F_Z_*	*F_X_*	*F_Z_*	*F_X_*	*F_Y_*
Force bearing point moving direction	*Z*	*Y*	*Z*	*X*	*Y*	*X*

**Table 2:** The sensor conditions in simulation.

Material	Structural Steel	Finite Element Type	Solid 186	Mesh Size	2 mm
*M_Y_* = 28 Nm	Supposing *F_Z_* 800 N, force bearing point moves 35 mm in *X* direction.				
Dimensions (mm^3^)	Sensor center plate		88 × 88 × 25		
	Horizontal beams	12 × 4 × 36	Vertical bemas	6 × 4 × 36	
Distance between the axis of the horizontal beam and the top surface of the workpiece (*H_H_*)					13 mm

**Table 3:** The stresses of the horizontal beams under *M_Y_*.

*H_H_* (mm)	3	5	7	9	11	13	15	17	19	21	23
*θ* (10^−30^)	4.459	4.481	4.495	4.507	4.516	4.525	4.533	4.539	4.541	4.539	4.530
*σ_r_* (10^−1^ MPa)	8.026	8.004	7.796	7.534	7.256	6.996	6.741	6.576	6.548	6.655	7.439

**Table 4:** Static performances of the sensor.

	Loading Point	1	2	3	4	5	6	7	8
*F_X_*	Linearity error (%)	−0.73	−0.64	−0.77	−0.65	0.96	−0.19	0.26	0.19
	Hysteresis error (%)	0.26	0.23	0.27	0.24	0.35	0.17	0.09	0.17
	Repeatability error (%)	0.76	0.40	0.39	0.25	0.60	0.71	0.52	0.81
*F_Y_*	Linearity error (%)	0.57	0.31	−0.72	−0.82	1.05	0.80	0.41	−0.34
	Hysteresis error (%)	0.21	0.11	0.26	0.30	0.38	0.29	0.15	0.38
	Repeatability error (%)	0.67	0.65	0.63	0.82	0.61	0.28	0.34	0.37
*F_Z_*	Linearity error (%)	−1.55	−0.48	−0.95	−1.30	−1.18	−0.84	−0.62	1.01
	Hysteresis error (%)	0.58	0.18	0.36	0.49	0.44	0.31	0.23	0.38
	Repeatability error (%)	0.66	0.62	0.46	0.81	0.43	0.60	0.56	0.61

**Table 5:** Milling parameters.

Test Groups	Test Series	Subtests	*a_p_* (mm)	*a_w_* (mm)	*v_f_* (mm/min)	*n* (rpm)	Workpiece Sizes (mm^3^)	Cutting Material
1	(1)	*A* and *B*	0.8	0.7	200	1200	88 × 88 × 20	AISI 1045
	(2)	*A* and *B*	1.6	0.7	200	1200		
	(3)	*A* and *B*	2.4	0.7	200	1200		
	(4)	*A* and *B*	3.2	0.7	200	1200		
	(5)	*A* and *B*	4.0	0.7	200	1200		
2	(1)	*A* and *B*	1	1.5	400	1600	88 × 88 × 20	Aluminum alloy 6061
	(2)	*A* and *B*	1	1.5	400	3200		
	(3)	*A* and *B*	1	1.5	400	4800		
	(4)	*A* and *B*	1	1.5	400	6400		
	(5)	*A* and *B*	1	1.5	400	8000		
3	(1)	*A* and *B*	2	1.5	400	2000	88 × 88 × 20	Aluminum alloy 6061
	(2)	*A* and *B*	2	1.5	500	2000		
	(3)	*A* and *B*	2	1.5	600	2000		
	(4)	*A* and *B*	2	1.5	700	2000		
	(5)	*A* and *B*	2	1.5	800	2000		

**Table 6:** The cross correlation coefficients (*R*) between each subtest result and the reference force arrays.

		Test Group 1			Test Group 2			Test Group 3		
		*F_X_*	*F_Y_*	*F_Z_*	*F_X_*	*F_Y_*	*F_Z_*	*F_X_*	*F_Y_*	*F_Z_*
*R*	Subtest *A*	0.9986	0.9823	0.9964	0.9865	0.9819	0.9569	0.9816	0.9780	0.9711
	Subtest *B*	0.9986	0.9878	0.9927	0.9511	0.9693	0.9666	0.9573	0.9763	0.9505

**Table 7:** The differences of the measured force between subtests *A* and *B* in different test groups.

	Test Series	Test Group 1			Test Group 2			Test Group 3		
		*F_X_*	*F_Y_*	*F_Z_*	*F_X_*	*F_Y_*	*F_Z_*	*F_X_*	*F_Y_*	*F_Z_*
Differences (%)	**(1)**	5.86	2.87	5.49	0.06	6.03	5.53	−4.76	−0.07	−3.68
	**(2)**	2.73	2.27	−2.26	6.14	3.27	−2.46	−1.36	2.97	4.46
	**(3)**	3.55	3.12	4.23	−6.29	−5.09	4.35	1.08	2.60	5.28
	**(4)**	3.70	3.66	3.59	−0.06	−4.51	−2.70	−1.46	−5.73	3.65
	**(5)**	−2.79	0.08	6.21	3.77	−2.83	5.14	−1.44	−1.32	−0.05

**Table 8:** Decoupling performance of related researches.

Related Researches	Forces	Decoupling Performances					
		Principal Components		Decoupling Components		Decoupling Results	
		Eccentric Range (*L_X_, L_Y_, L_Z_*) (mm)	Output Error (%)	Eccentric Range (*L_X_, L_Y_, L_Z_*) (mm)	Output Error (%)	Eccentric Range (*L_X_, L_Y_, L_Z_*) (mm)	Meaured Error (%)
[15]	*F_X_*	(50, 50, 50)	0.18	/	/	/	/
	*F_Y_*		0.18				
	*F_Z_*		1.28				
	*F^1^*	/	/	/	/	(0, 0, 0)	1.49 (static test)
[21]	*F_X_*	/	/	(8, 12, 31)	4.74, 4.09,^2^	/	/
	*F_Y_*				2.58, 5.38		
	*F_Z_*				7.33,10.51		
[17,18,19]	*F_X_*	/	/	/	/	(10, 10, <20)	8–9,^3^
	*F_Y_*						20–35
	*F_Z_*						60–90 (milling test)
[16]	*F_X_*	(35, 35, 15)	≤5.2	(35, 35, 15)	≤3.94	(35, 35, 15)	≤4.80
	*F_Y_*		≤5.6		≤2.98		≤4.58
	*F_Z_*		≤4.8		≤3.48		≤4.87 (static test)
This work	*F_X_*	(35, 35, 15)	≤3.36	(35, 35, 15)	≤2.42	(35, 35, 15)	≤4.50
	*F_Y_*		≤2.06		≤2.70		≤4.58
	*F_Z_*		≤3.50		≤3.20		≤4.52 (static test)
	*F*	/	/	/	/	(35, 35, 15)	≤4.07

^1^ *F* stands for the resultant force of *F_X_*, *F_Y_* and *F_Z_*. ^2^ Based on the explanation in the paper, the difference of coupling error on symmetrical elastic beams to *Z* axis (10.51% − 7.33% = 3.18%) is mostly caused by the eccentric distance of the tool tip (i.e., *L_X_* − *L_Y_* = 4 mm). ^3^ The errors were obtained via high speed milling test without eccentric compensations of additional moments.

**Table 9:** Sensor resonance frequency and sensitivity in related researches.

Elastic Beams	[15]	[21]	[17,18,19]	[16]	
Structure	Octagonal rings		Parallel vertical beams	Cross beam	This work
Material	AISI 4140	AISI 1045	6061	AISI 630	
Sensor type	Milling table sensor	Turning sensor	Milling table sensor		
Sensor resonance frequency					
Upper plate					
size (mm^3^)	245 × 270 × 25	/	48 × 48 × 4	88 × 88 × 20	88 × 88 × 25
Material	Unknown	/	6061	AISI 630	AISI 630
Clamp					
Dimensions (mm^3^)	/	16 ×16 ×100 (turning tool)	/	88 × 88 × 15 (workpiece)	88 × 88 × 20 (workpiece)
Material	/	Steel	/	AISI 1045	AISI 1045
Resonance frequency (Hz)	≥1200	≥1122	≥9106	≥680	≥1663.5
Sensor sensitivity					
Circuit amplification	Unknown	1	1	1	1
Output sensitivity *F_X_*, *F_Y_*, *F_Z_* (10^-3^ mV/N/V)	3.61	1.06	0.088	1.72	2.68
	3.47	1.14	0.154	1.71	2.67
	1.81 (Static test)	0.18 (Static test)	0.105 (Dynamic test)	12.50 (Static test)	2.16 (Static test)
